# Bilateral Painless Vision Loss From Probable Leptomeningeal Metastasis During Systemic Response in Esophageal Adenocarcinoma

**DOI:** 10.7759/cureus.115922

**Published:** 2026-09-07

**Authors:** Zoe West, Amber Cano, Jay Patel, Darshil Shah

**Affiliations:** 1 Gastrointestinal Oncology, Ironwood Cancer &amp; Research Centers, Goodyear, USA; 2 Neuro-Ophthalmology, Barrow Neurological Institute, Phoenix, USA

**Keywords:** cerebrospinal fluid cytology, esophageal adenocarcinoma, gastrointestinal oncology, leptomeningeal carcinomatosis, leptomeningeal metastasis, neuro-ophthalmology, optic disc edema, optic nerve sheath enhancement, sanctuary site, signet ring cell

## Abstract

Leptomeningeal metastasis (LM) is a rare complication of esophageal adenocarcinoma, with reported cases typically presenting with meningitic, encephalopathic, or multifocal neurologic symptoms. We report a 70-year-old man with metastatic esophageal adenocarcinoma with signet-ring cell features who developed subacute, painless, bilateral visual loss with bilateral optic disc edema as the initial manifestation of probable LM. Symptoms arose four months into first-line folinic acid, fluorouracil, and oxaliplatin (FOLFOX) plus pembrolizumab while all measurable extracranial disease was responding and in the absence of headache, meningismus, or other neurologic symptoms. Contrast-enhanced MRI demonstrated bilateral optic nerve sheath enhancement with subtle linear leptomeningeal enhancement over the cerebral convexities and no parenchymal brain metastases. Over the ensuing days, the patient developed urinary incontinence, gait instability, lower-extremity weakness, and cognitive decline. Cerebrospinal fluid analysis showed markedly elevated protein, lymphocytic pleocytosis, normal glucose, and rare atypical cells suspicious for malignancy; flow cytometry and cultures were negative. Although CSF cytology was not definitively diagnostic, the combined clinical presentation, characteristic MRI findings, and supportive CSF abnormalities in the setting of known metastatic malignancy were consistent with probable LM. Whole-brain radiotherapy was initiated but discontinued after one fraction because of rapid neurologic deterioration, and the patient died four weeks after the diagnosis. This case highlights an unusual presentation of probable LM in esophageal adenocarcinoma, with subacute painless bilateral visual loss and optic disc edema preceding other neurologic manifestations despite concurrent extracranial disease response.

## Introduction

Leptomeningeal metastasis (LM) is the dissemination of malignant cells within the leptomeninges and subarachnoid space and complicates approximately 5% of advanced solid tumors, most commonly breast cancer, lung cancer, and melanoma [1]. In gastroesophageal carcinoma, it is rare, and published experience in esophageal adenocarcinoma consists largely of isolated case reports and a small institutional series [2,3]. Reported presentations generally reflect the classic multifocal syndrome of leptomeningeal involvement, including headache, neck stiffness, nausea, vomiting, confusion, ataxia, dizziness, cranial neuropathies, and radicular symptoms [4-10].

Diagnosis can be challenging because CSF cytology may be negative or equivocal on initial sampling, while contrast-enhanced MRI findings may overlap with infectious and inflammatory processes [11,12]. Current European Association of Neuro-Oncology-European Society for Medical Oncology (EANO-ESMO) criteria therefore allow a diagnosis of probable LM when compatible clinical and imaging findings occur in a patient with known malignancy despite nondiagnostic cytology [11-13].

Ocular manifestations of leptomeningeal disease are well recognized and may occasionally herald the diagnosis. Optic nerve involvement may manifest as optic neuropathy, optic disc edema, and optic nerve or perineural enhancement on contrast-enhanced MRI [14]. In a systematic review of 41 patients with ocular involvement, ocular symptoms were present at diagnosis in 83%, were the sole manifestation in 7%, and visual loss was the most common ocular symptom [14]. Visual loss has also been reported as a presenting manifestation of leptomeningeal disease from gastrointestinal primaries [15]. However, among previously reported cases of LM from esophageal adenocarcinoma reviewed for this report, presentations were predominantly meningitic, encephalopathic, or otherwise neurologically multifocal. One prior case included vision loss, but this occurred in association with headache and falls rather than as an isolated visual presentation. We identified no prior report of bilateral painless vision loss as the dominant initial manifestation. We therefore describe this unusual presentation of probable LM, which developed four months into first-line chemoimmunotherapy despite a concurrent response in all measurable extracranial disease.

## Case presentation

A 70-year-old male with gastroesophageal reflux disease, hypothyroidism, and prior tobacco use presented with a two-month history of progressive dysphagia and odynophagia. Upper endoscopy demonstrated a partially obstructing circumferential mass in the lower third of the esophagus, and biopsy confirmed esophageal adenocarcinoma with signet-ring cell features (Figure 1).

**Figure 1:**
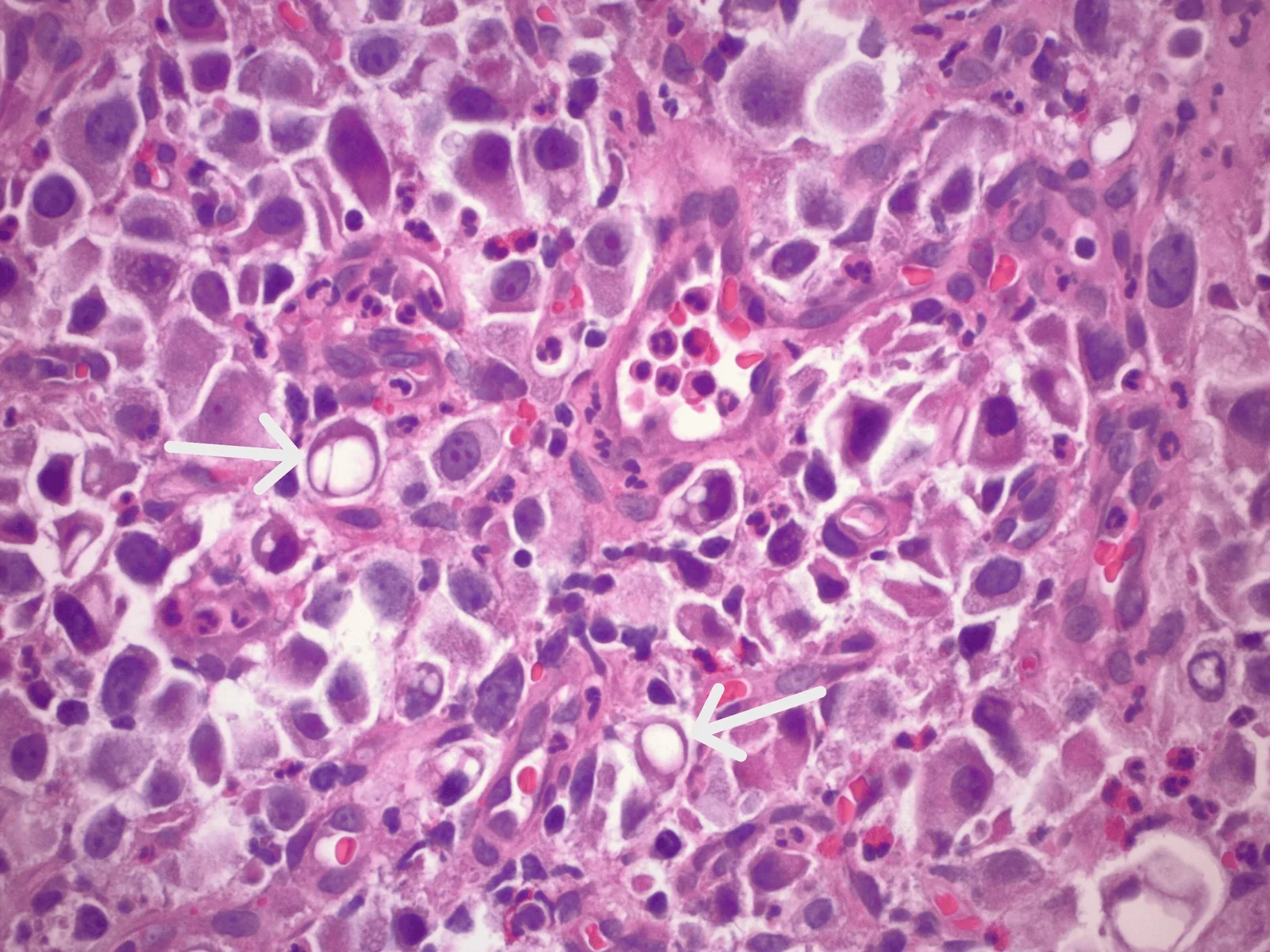
Esophageal adenocarcinoma with signet-ring cell features Hematoxylin and eosin-stained esophageal biopsy demonstrating adenocarcinoma with signet-ring cell features. White arrows indicate representative signet-ring cells characterized by intracytoplasmic mucin vacuoles with eccentrically displaced nuclei.

Staging fluorine-18 fluorodeoxyglucose positron emission tomography/computed tomography (18F-FDG PET/CT) demonstrated an avid distal esophageal primary tumor, metastatic supraclavicular, mediastinal, and right retrocrural lymphadenopathy, sclerotic thoracolumbar bone metastases, and diffuse enlargement and hypermetabolism of the left iliopsoas muscle concerning for metastatic infiltration (Figure 2).

**Figure 2:**
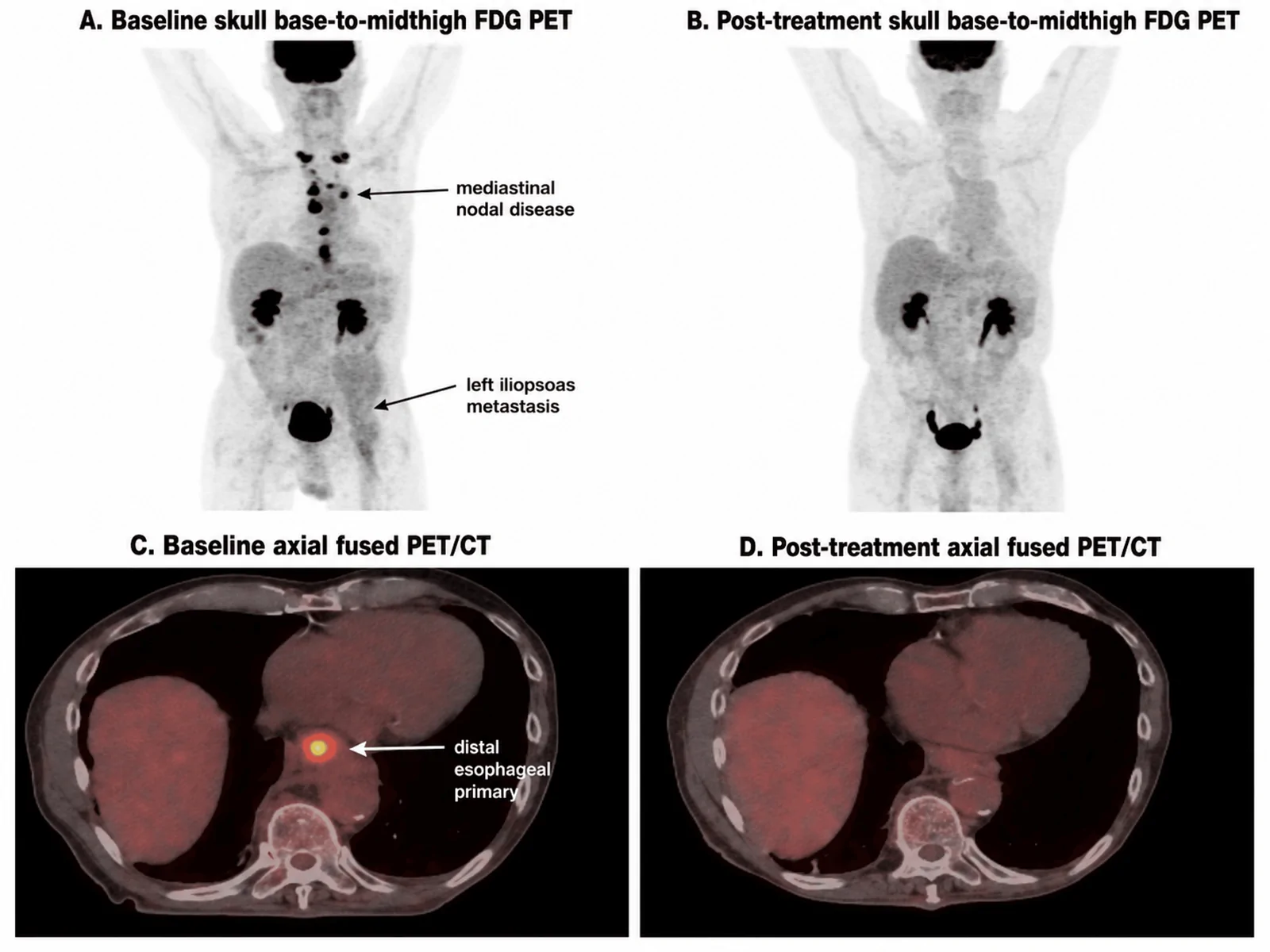
Baseline and post-treatment FDG PET/CT demonstrating marked systemic response (A) Baseline skull base-to-midthigh FDG PET demonstrating hypermetabolic mediastinal nodal disease and left iliopsoas metastasis. (B) Post-treatment skull base-to-midthigh FDG PET demonstrating marked interval reduction in previously hypermetabolic systemic disease. (C) Baseline axial fused PET/CT demonstrating the hypermetabolic distal esophageal primary. (D) Post-treatment axial fused PET/CT demonstrating marked interval decrease in FDG uptake at the distal esophageal primary following chemoimmunotherapy. FDG PET/CT: fluorodeoxyglucose positron emission tomography/computed tomography

Biopsies of a T11 vertebral lesion and the left psoas muscle confirmed metastatic adenocarcinoma consistent with an esophageal primary. The tumor was human epidermal growth factor receptor 2 (HER2) negative, mismatch repair proficient (pMMR), and programmed death-ligand 1 (PD-L1) positive, with a combined positive score (CPS) of 5. Baseline brain imaging was not performed because the patient had no neurologic symptoms and routine central nervous system (CNS) staging is not indicated in esophageal adenocarcinoma. First-line chemoimmunotherapy with folinic acid, fluorouracil, and oxaliplatin (FOLFOX) plus pembrolizumab was initiated. The patient achieved an excellent systemic partial response after six cycles, assessed at approximately three months, with ongoing extracranial disease control and improvement in dysphagia.

Approximately four months after initiation of chemoimmunotherapy, the patient developed subacute, painless, bilateral visual decline, first noted as blurring of the superior visual field in both eyes, without headache, diplopia, neck stiffness, nausea, weakness, or cognitive change. Over the ensuing days, the deficit became constant and progressive, and was consistently worse in the right eye. At its worst, best-corrected visual acuity was 20/400 in the right eye and 20/50 in the left eye.

Neuro-ophthalmologic examination demonstrated dyschromatopsia (0/8 color plates in the right eye) and a right relative afferent pupillary defect. Automated perimetry (Humphrey 24-2C, Carl Zeiss Meditec AG, Jena, Germany) demonstrated profound generalized visual-field depression in the right eye (mean deviation −31.63 dB) and marked visual-field depression in the left eye (mean deviation −19.33 dB). Dilated fundoscopy revealed circumferential (360°) optic disc edema with peripapillary hemorrhages in both eyes. Optical coherence tomography demonstrated marked bilateral retinal nerve fiber layer thickening. Extraocular movements were full, and the remainder of the cranial nerve and neurologic examination was normal.

Contrast-enhanced MRI of the brain and orbits demonstrated bilateral, nearly symmetric optic nerve sheath enhancement involving the intraorbital and intracanalicular segments, greater on the left, with minimal associated T2 hyperintensity and no optic chiasm involvement (Figure 3). Subtle linear leptomeningeal enhancement was also present along the right temporal and parietal lobes and the high left frontal convexity. No parenchymal mass lesion, hydrocephalus, or acute ischemia was identified.

**Figure 3:**
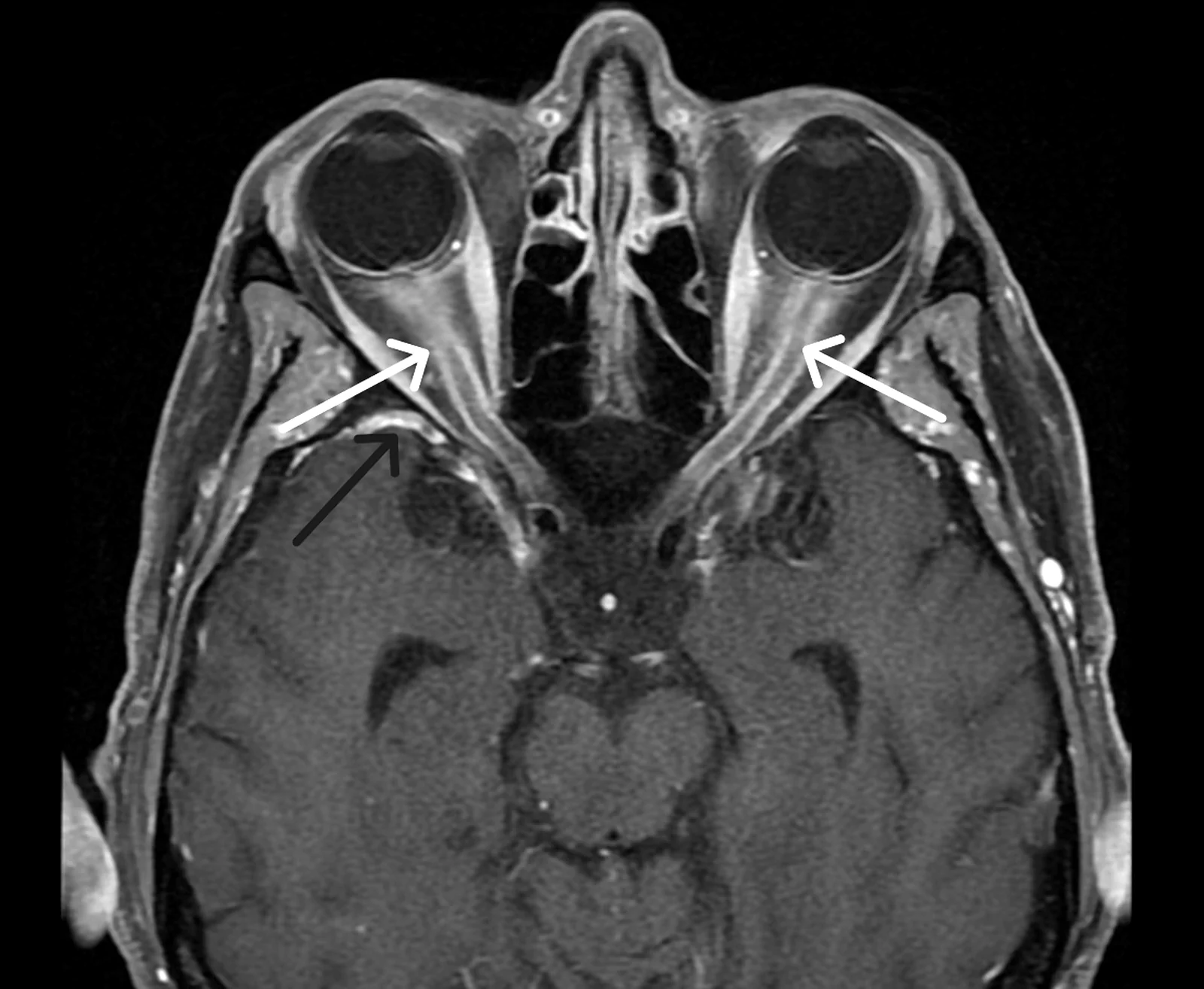
Bilateral optic nerve sheath enhancement with adjacent right temporal leptomeningeal involvement Axial post-contrast fat-saturated MRI of the orbits demonstrating bilateral optic nerve sheath enhancement, nearly symmetric but greater on the left (white arrows), with additional enhancement along the right anterior temporal lobe (black arrow), supportive of leptomeningeal involvement.

The differential diagnosis included leptomeningeal involvement, inflammatory optic neuropathy including checkpoint inhibitor-associated optic neuritis or optic perineuritis, granulomatous disease, paraneoplastic optic neuropathy, and infection [16]. Checkpoint inhibitor-associated optic neuritis was an important competing diagnosis given ongoing pembrolizumab therapy. Empiric high-dose dexamethasone (10 mg orally every six hours for four days) was initiated while further evaluation proceeded, with transient improvement in vision. However, over the ensuing days, the patient developed urinary incontinence, gait instability, lower-extremity weakness, and cognitive decline.

Lumbar puncture demonstrated turbid CSF with a protein level of 264 mg/dL, mild lymphocytic pleocytosis, and normal glucose. Opening pressure was not measured. Cytology revealed rare atypical cells suspicious for malignancy (Figure 4).

**Figure 4:**
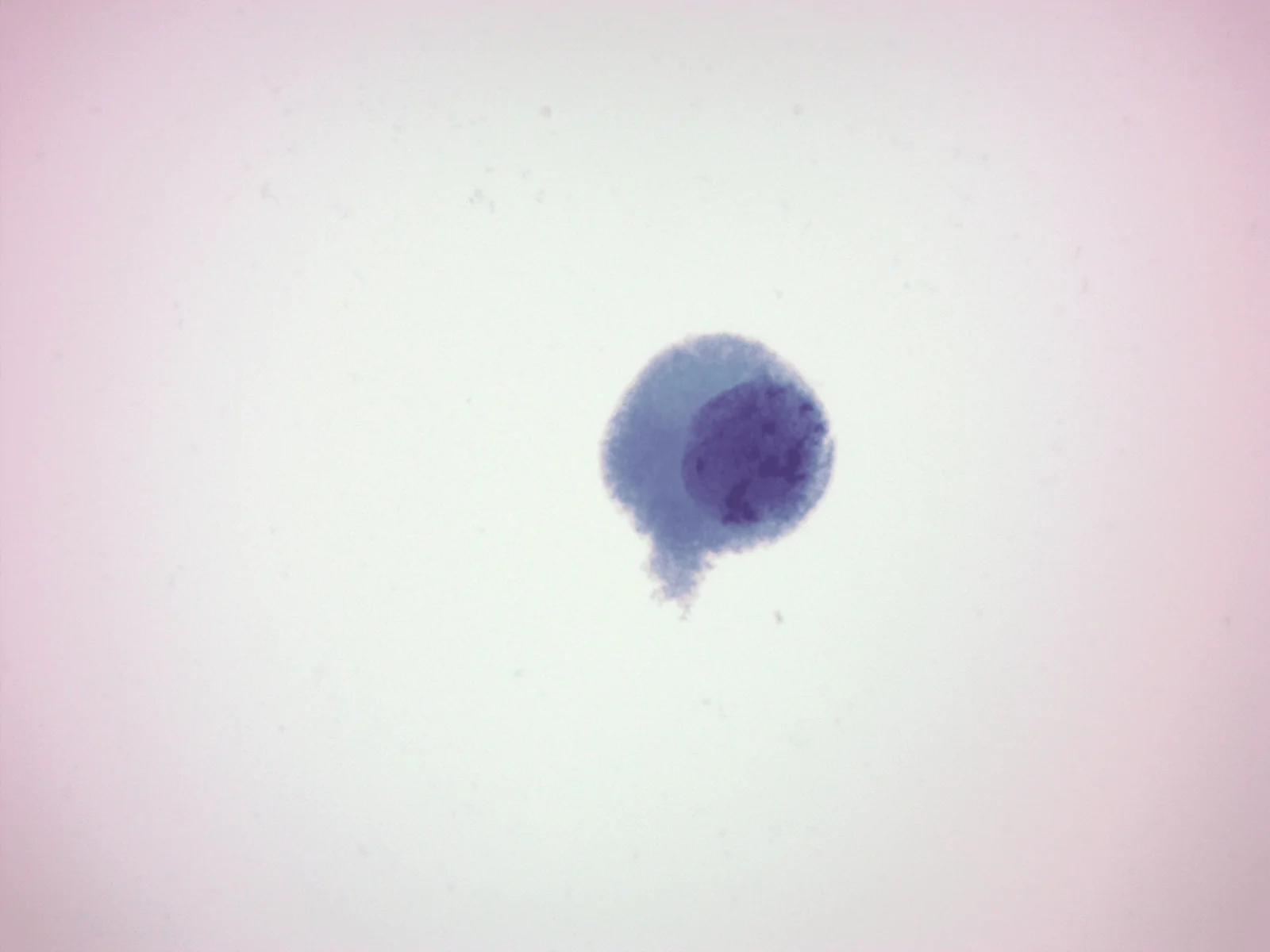
CSF cytology revealed rare atypical cells suspicious for malignancy Cerebrospinal fluid (CSF) cytology demonstrates a rare atypical cell with an enlarged, irregular nucleus and increased nuclear-to-cytoplasmic ratio. The finding is suspicious for malignancy but not definitively diagnostic of leptomeningeal metastasis.

Cellularity was insufficient for cell-block preparation and immunohistochemical characterization; flow cytometry and cultures were negative. Although CSF cytology was not definitive, the combination of a progressive multifocal neurologic syndrome, bilateral optic nerve sheath and intracranial leptomeningeal enhancement on MRI, abnormal CSF findings, and known metastatic adenocarcinoma was considered consistent with probable LM.

Chemoimmunotherapy was discontinued. Whole-brain radiotherapy (30 Gy in 10 fractions) was initiated but discontinued after the first fraction because of continued neurologic deterioration. Intrathecal therapy was not pursued because of poor performance status and rapid clinical decline. The patient transitioned to comfort-focused care with hospice support and died four weeks after the diagnosis of probable LM.

## Discussion

LM from esophageal adenocarcinoma is rare and carries a poor prognosis, with survival typically measured in weeks [2,3]. Although ocular manifestations are recognized, this case is notable for bilateral painless visual loss as the dominant initial manifestation. Previously reported cases have more commonly presented with headache, meningismus, nausea, vomiting, confusion, ataxia, or dizziness [4-10,17] (Table 1).

**Table 1:** Reported cases of leptomeningeal metastasis (LM) in esophageal adenocarcinoma Representative published cases; not intended as a systematic review.

Reference	Age/sex	Presentation	Treatment	Survival from LM diagnosis
Abdo et al. [5]	50/M	Confusion, neck stiffness	Corticosteroids	~8 weeks
Goddeeris et al. [6]	60/M	Confusion, ataxia	Supportive care	7 weeks
Goddeeris et al. [6]	46/M	Headache, vomiting	Corticosteroids	~3 weeks
Reich DS [7]	50/M	Vision loss, headache, falls	Whole-brain radiotherapy, intrathecal chemotherapy	Several weeks
Ahmed et al. [8]	47/F	Headache, neck stiffness, nausea	Corticosteroids, radiotherapy	5 weeks
Fresko et al. [9]	47/M	Headache, neck stiffness	Supportive care	1 week
Boursiquot et al. [17]	76/M	Delirium, hyponatremia	Intrathecal methotrexate	6 weeks
Cunha et al. [10]	51/M	Hypoacusis, nausea, and loss of balance	Supportive care	~6 weeks
Grabill et al. [4]	64/M	Dizziness, ataxia, nausea, vomiting	Supportive care, hospice	~3 weeks
Present case	70/M	Bilateral painless vision loss, optic disc edema	Whole-brain radiotherapy (1 fraction)	4 weeks

In contrast, our patient presented with isolated bilateral painless visual loss with optic disc edema and no other neurologic symptoms; a multifocal neurologic syndrome developed over the ensuing days. Leptomeningeal disease in gastroesophageal cancer may therefore present without the meningeal or encephalopathic features that typically prompt cerebrospinal fluid evaluation.

Leptomeningeal and optic nerve sheath involvement likely accounted for the bilateral optic neuropathy through direct infiltration of the perioptic subarachnoid space. This was supported by perioptic and intracranial leptomeningeal enhancement on MRI and by cerebrospinal fluid demonstrating pleocytosis, markedly elevated protein, and rare atypical cells suspicious for malignancy. The associated bilateral optic disc edema was most consistent with an infiltrative optic neuropathy; however, a contribution from elevated intracranial pressure could not be excluded because CSF opening pressure was not measured. Checkpoint inhibitor-associated optic neuritis or optic perineuritis, as well as other inflammatory, granulomatous, or paraneoplastic optic neuropathies, were considered in the differential diagnosis. However, the presence of intracranial leptomeningeal enhancement extending beyond the orbits, subsequent progressive multifocal neurologic deterioration, and supportive CSF abnormalities favored leptomeningeal disease over an isolated inflammatory or paraneoplastic optic neuropathy. CSF cultures were negative. In patients with malignancy and optic nerve sheath enhancement, assessment for additional intracranial meningeal enhancement may therefore provide an important diagnostic clue.

CSF cytology was suspicious for malignancy but not definitively diagnostic of LM, and cellularity was insufficient for confirmatory immunohistochemistry. Repeat CSF sampling, which can improve diagnostic sensitivity after equivocal cytology, was not feasible because of the patient’s rapid clinical decline [11,14]. In the setting of known metastatic adenocarcinoma, the compatible progressive neurologic syndrome, characteristic perioptic and intracranial leptomeningeal enhancement on MRI, and supportive CSF abnormalities were consistent with probable LM under the EANO-ESMO framework [12]. Additional inflammatory evaluation and visual evoked potentials were not obtained during the rapidly progressive clinical course.

The tumor also demonstrated signet-ring cell features, as did a recently reported case of esophageal adenocarcinoma with LM [4]. Whether poorly cohesive histology predisposes to leptomeningeal dissemination is unknown, but this observation may warrant further study as additional cases accumulate.

More broadly, this case is consistent with a pattern of CNS sanctuary-site progression, in which extracranial disease control may coexist with progression behind the blood-brain and blood-CSF barriers [18]. In this patient, extracranial disease was responding at every measured site when leptomeningeal disease emerged, providing neither clinical warning nor protection from CNS progression. As chemoimmunotherapy extends survival in advanced gastroesophageal cancer, clinicians may increasingly encounter such discordant patterns of progression. Recognition is important because new neurologic or visual symptoms warrant prompt CNS evaluation even when systemic disease remains controlled. Treatment remains palliative and may include radiotherapy, intrathecal therapy, CNS-active systemic therapy when appropriate, and supportive care [1,11,17].

## Conclusions

Bilateral painless visual loss with optic disc edema may herald leptomeningeal metastasis (LM) in esophageal adenocarcinoma, even in the absence of headache, meningismus, or parenchymal brain metastases, and while extracranial disease is responding. Evaluation should include neuroimaging beyond the orbits: optic nerve sheath enhancement, particularly when accompanied by meningeal enhancement over the cerebral convexities, should raise suspicion for leptomeningeal spread and prompt cerebrospinal fluid evaluation. When cytology is equivocal, probable LM may be supported by the combination of a compatible clinical presentation, characteristic imaging findings, and an abnormal cerebrospinal fluid profile in a patient with known malignancy; repeat lumbar puncture should be considered when clinically feasible. Systemic disease control does not exclude the possibility of central nervous system progression.
